# Supplementary material for: Sequencing and Comparative Analysis of the Chloroplast Genome of Angelica polymorpha and the Development of a Novel Indel Marker for Species Identification
Source: Molecules. 2019 Mar 15;24(6):1038. doi: 10.3390/molecules24061038 (PMC6471784; doi:10.3390/molecules24061038)
Supplement: Supplementary file 1 [file molecules-24-01038-s001.pdf]

molecules

Supplementary Materials: Sequencing and Comparative Analysis of the Chloroplast Genome of *Angelica polymorpha* and the Development of a Novel Indel Marker for Species Identification

Inkyu Park, Sungyu Yang, Wook Jin Kim, Jun-Ho Song, Hyun-Sook Lee, Hyun Oh Lee, Jung-Hyun Lee, Sang-Nag Ahn, and Byeong Cheol Moon*

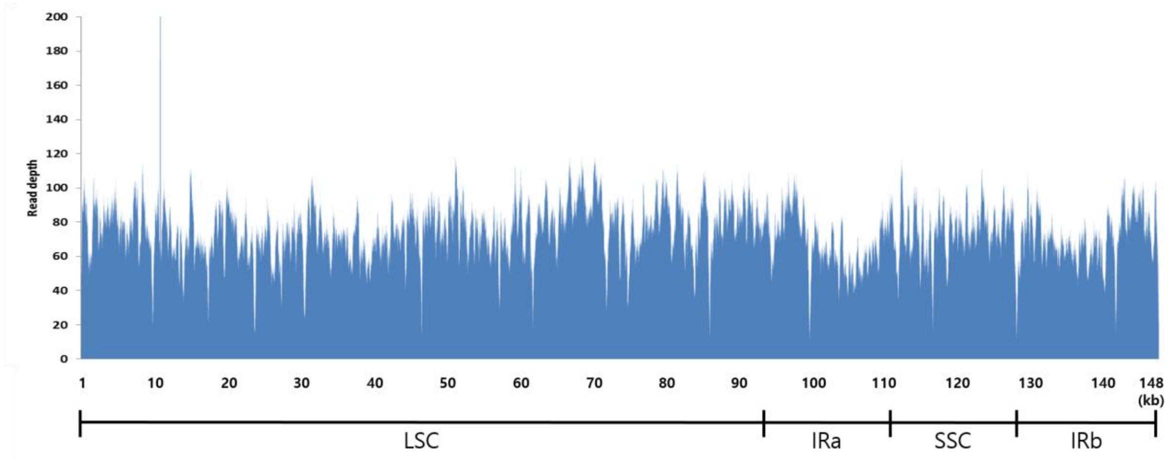

Figure S1: Distribution of paired-end reads mapped onto the complete chloroplast (CP) genome sequence of *A. polymorpha*.

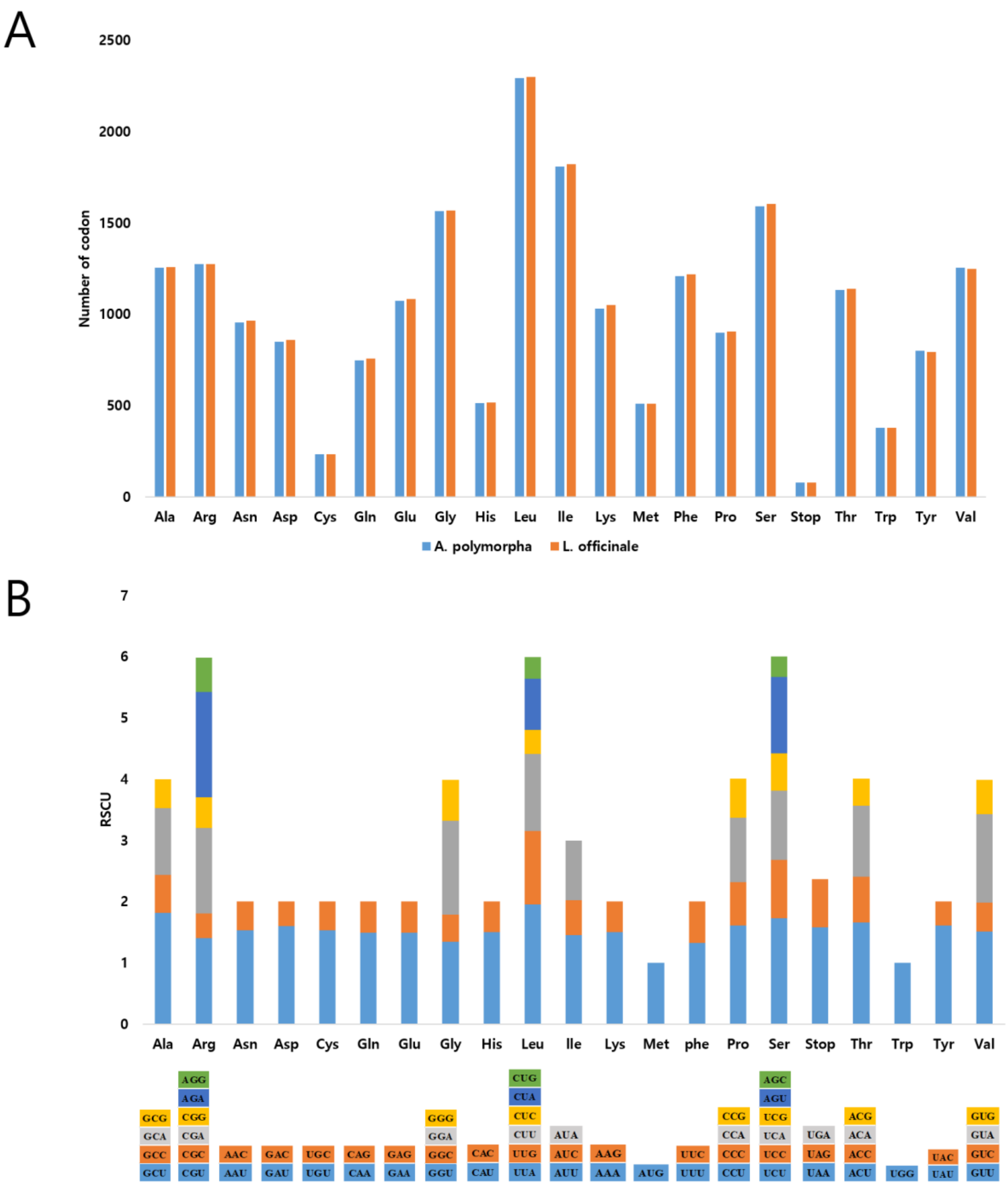

Figure S2: Codon frequencies and relative synonymous codon usage (RSCU) values of the CP genomes of *A. polymorpha* and *L. officinale*. (A) Amino acid frequencies in protein-coding genes. (B) Codon usage for 20 amino acids and stop codons in 78 protein-coding genes.

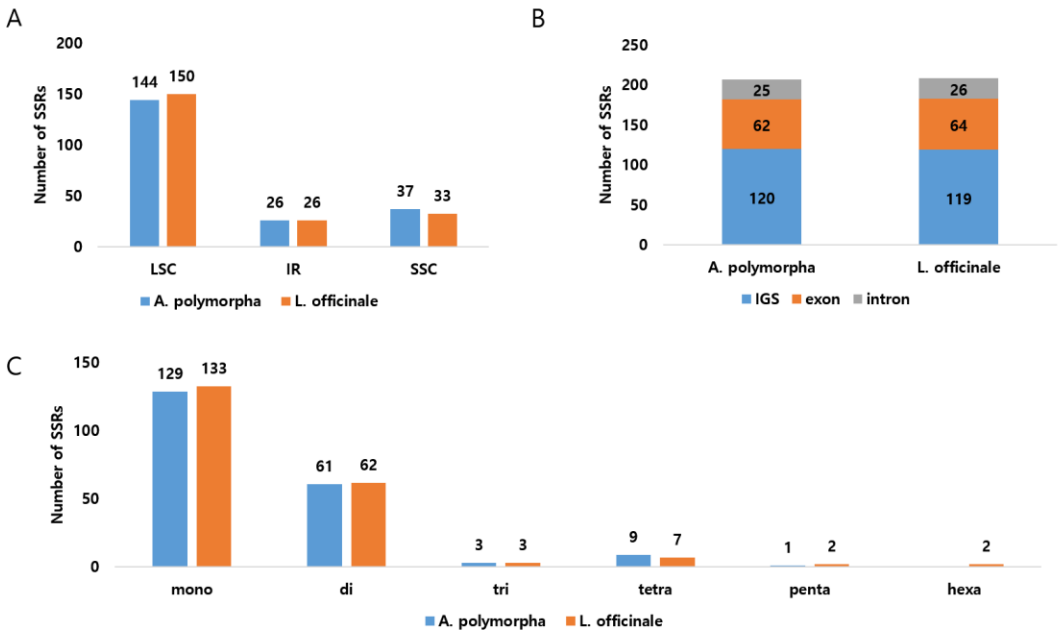

14
15 **Figure S3:** Distribution of simple sequence repeats (SSRs) in the CP genomes of *A. polymorpha* and *L. officinale*.
16 (A) Number of SSRs in CP genomes. (B) Number of SSRs in exons, introns and intergenic spacer (IGS) regions.
17 (C) Number of different types of SSRs in CP genomes.

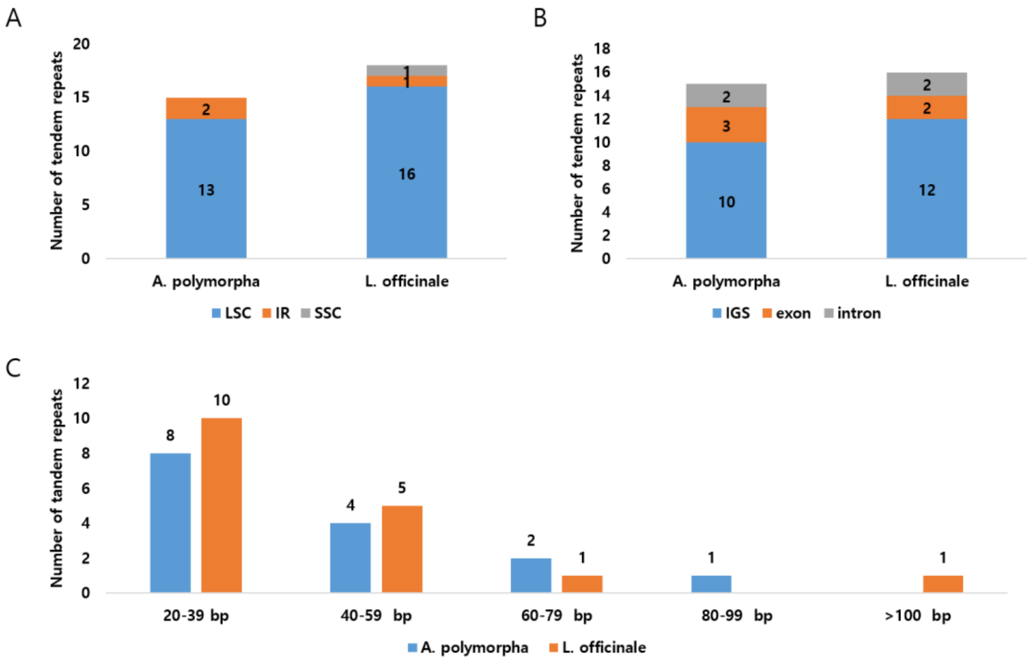

Figure S4: Analysis of tandem repeats in the CP genomes of *A. polymorpha* and *L. officinale*. (A) Distribution of tandem repeats in different regions of CP genomes. (B) Number of tandem repeats in IGS regions, exons, and introns. (C) Distribution of tandem repeats of variable lengths in CP genomes.

Table S1: Details of raw sequence reads and CP genome assembly of *A. polymorpha*.

**Parameter**	**Number of reads**
Input reads	5,000,000
Trimmed reads	4,307,210
Total raw bases (bp)	1,255,000,000
Trimmed bases (bp)	940,906,215
Aligned reads	51,266
Coverage (X)	75
Chloroplast genome size (bp)	147,127

Table S2: PCR-based sequence validation of junctions between the large single copy (LSC), small single copy (SSC), and inverted repeat (IRa and IRb) regions in the CP genome of *A. polymorpha*.

Location	PCR-based sequence (bp)	Start position (bp)	End position (bp)	Identity (%)
LSC/IRa	376	93,336	93,711	100
IRa/SSC	175	111,453	111,572	100
SSC/IRb	508	128,855	129,362	100
IRb/LSC	732	146,942	546	100

Table S3: List of genes and encoded proteins identified in the CP genomes of *A. polymorpha* and *L. officinale*.

Gene group	Gene name
Photosystem I	*psaA, B, C, I, J, ycf3*[2)]*, ycf4*
Photosystem II	*psbA, B, C, D, E, F, H, I, J, K, L, M, N, T, Z*
Cytochrome b6/f	*petA, B*[1)]*, D*[1)]*, G, L, N*
ATP synthase	*atpA, B, E, F*[1)]*, H, I*
Rubisco	*rbcL*
NADH oxidoreductase	*ndhA*[1)]*, B*[1)3)]*, C, D, E, F, G, H, I, J, K*
Large subunit ribosomal proteins	*rpl2*[1)3)]*, 14, 16*[1)]*, 20, 22, 23*[3)]*, 32, 33, 36*
Small subunit ribosomal proteins	*rps2, 3, 4, 7*[3)]*, 8, 11, 12*[2)3)4)]*, 14, 15, 16, 18, 19*
RNA polymerase	*rpoA, B, C1*[1)]*, C2*
Unknown function protein-coding gene	*ycf1*[3)]*, 2, 15*[3)]
Other genes	*accD, ccsA, cemA, clpP*[2)]*, matK*
Ribosomal RNAs	*rrn16*[3)]*, 23*[3)]*, 4.5*[3)]*, 5*[3)]
Transfer RNAs	*trnA-UGC*[1)3)]*, trnC-GCA, trnD-GUC, trnE-UUC, trnF-GAA, trnG-UCC*[1)]*, trnG-GCC, trnH-GUG, trnI-CAU, trnI-GAU*[1)3)]*, trnK-UUU*[1)]*, trnL-UAA*[1)]*, trnL-UAG, trnL-CAA*[3)]*, trnM-CAU, trnfM-CAU, trnN-GUU*[3)]*, trnP-UGG, trnQ-UUG, trnR-ACG*[3)]*, trnR-UCU, trnS-GCU, trnS-GGA, trnS-UGA, trnT-GGU, trnT-UGU, trnV-UAC*[1)]*, trnV-GAC*[3)]*, trnW-CCA, trnY-GUA*

[1] Genes containing a single intron.

[2] Genes containing two introns.

[3] Genes present as two copies in the IR regions.

[4] Trans-spliced gene.

Table S4: List of intron-containing genes in the CP genomes of *A. polymorpha* and *L. officinale*.

No.	Gene	Region	Exon I[1]	Intron I[1]	Exon II[1]	Intron II[1]	Exon III[1]
1	*trnK-UUU*	LSC	37	2523*	35		
2	*rps16*	LSC	40	855 (857)	197		
3	*trnG-UCC*	LSC	23	704	48		
4	*atpF*	LSC	145	722	401		
5	*rpoC1*	LSC	435	754	1605		
6	*ycf3*	LSC	126	720	226	778	155
7	*trnL-UAA*	LSC	35	504	50		
8	*trnV-UAC*	LSC	39	591	35		
9	*rps12**	LSC	114		232		26
10	*clpP*	LSC	71	840	292	638	231
11	*petB*	LSC	6	756	642		
12	*petD*	LSC	8	743	475		
13	*rpl16*	LSC	9	969	399		
14	*ndhB*	IR	777	684	756		
15	*trnI-GAU*	IR	37	951	35		
16	*trnA-UGC*	IR	38	817	35		
17	*ndhA*	SSC	553	1073	540		

[1] Numbers represent the gene size (bp) in *A. polymorpha*; numbers in parentheses represent the gene size in *L. officinale*. * The intron length of *trnK-UUU* includes *matK*.

Table S5: Codon-anticodon recognition patterns and codon usage in the CP genomes of *A. polymorpha* and *L. officinale*.

Amino acid	Codon	*A. polymorpha*		tRNA
		Count	RSCU[1]	
Phe	UUU	798	1.32	
Phe	UUC	411	0.68	trnF-GAA
Leu	UUA	749	1.96	trnL-UAA
Leu	UUG	450	1.18	trnL-CAA
Leu	CUU	483	1.26	
Leu	CUC	154	0.4	
Leu	CUA	316	0.83	trnL-UAG
Leu	CUG	141	0.37	
Ile	AUU	875	1.45	
Ile	AUC	340	0.56	trnI-GAU
Ile	AUA	595	0.99	trnI-CAU<br>trn(f)M-CAU<br>trnM-CAU
Met	AUG	512	1	trnM-CAU
Val	GUU	474	1.51	
Val	GUC	147	0.47	trnV-GAC
Val	GUA	458	1.46	trnV-UAC
Val	GUG	176	0.56	
Ser	UCU	463	1.75	
Ser	UCC	251	0.95	trnS-GGA
Ser	UCA	294	1.11	trnS-UGA
Ser	UCG	167	0.63	
Pro	CCU	354	1.57	
Pro	CCC	163	0.72	trnS-GCU
Pro	CCA	237	1.05	
Pro	CCG	146	0.65	
Thr	ACU	467	1.65	trnP-UGG
Thr	ACC	210	0.74	
Thr	ACA	333	1.18	
Thr	ACG	123	0.43	trnT-GGU
Ala	GCU	569	1.81	trnT-UGU
Ala	GCC	198	0.63	
Ala	GCA	341	1.09	
Ala	GCG	147	0.47	trnG-UCC
Tyr	UAU	644	1.61	trnA-UGC

Tyr	UAC	157	0.39	
Stop	UAA	43	1.61	
Stop	UAG	21	0.79	trnY-GUA
His	CAU	389	1.51	
His	CAC	126	0.49	
Gln	CAA	558	1.49	
Gln	CAG	191	0.51	
Asn	AAU	722	1.51	trnH-GUG
Asn	AAC	233	0.49	trnQ-UUG
Lys	AAA	774	1.5	
Lys	AAG	256	0.5	
Asp	GAU	677	1.59	trnN-GUU
Asp	GAC	172	0.41	trnK-UUU
Glu	GAA	801	1.49	
Glu	GAG	273	0.51	
Cys	UGU	175	1.51	trnD-GUC
Cys	UGC	57	0.49	trnE-UUC
Stop	UGA	16	0.6	
Trp	UGG	377	1	
Arg	CGU	298	1.4	trnC-GCA
Arg	CGC	87	0.41	trnW-CCA
Arg	CGA	299	1.41	trnR-ACG
Arg	CGG	109	0.51	
Ser	AGU	327	1.23	
Ser	AGC	89	0.34	
Arg	AGA	359	1.69	trnR-UCU
Arg	AGG	124	0.58	
Gly	GGU	530	1.35	
Gly	GGC	176	0.45	trnG-GCC
Gly	GGA	591	1.51	trnG-UCC
Gly	GGG	268	0.68	

[1] RSCU, relative synonymous codon usage.

Table S6: Details of palindromic repeats present in the LSC region of the CP genomes of *A. polymorpha* and *L. officinale*.

Species	Position	Loop (bp)	Position	Repeat unit length (bp)	Repeat unit sequence
A. polymorpha	IGS[1] *(trnH-GUG, psbA)*	6	IGS *(rnH-GUG, psbA)*	31	TGAAATATAAAAAGAAGAAATACCGCCCTCTTG
	IGS *(psbM, trnD-GUC)*	490	IGS *(trnE-UUC, trnT-GGU)*	39	AAAAGGGAAAGATGATTGATGTACTTATTGAATCTGTCG
	IGS *(trnT-GGU, psbD)*	62	IGS *(trnT-GGU, psbD)*	26	TTATCATCCTACTAAATTAGAATTTA
	IGS *(psbE, petL)*	0	IGS *(psbE, petL)*	32	TCTTTCTTACTTTACTTGTTGCTTGTCAGTGT
	IGS *(psbT, psbN)*	3	IGS *(psbT, psbN)*	21	TTGAAGTAATGAGTCCCCCAA
L. officinale	IGS *(trnH-GUG, psbA)*	13	IGS *(rnH-GUG, psbA)*	24	AAAAAAAGCAATACCGCCCTCTTG
	IGS *(psbM, trnD-GUC)*	490	IGS *(trnE-UUC, trnT-GGU)*	39	AAAAGGGAAAGATGATTGATGTACTTATTGAATCTGTCG
	IGS *(trnT-GGU, psbD)*	62	IGS *(trnT-GGU, psbD)*	26	TTATCATCCTACTAAATTAGAATTTA
	IGS *(psbE, petL)*	10	IGS *(psbE, petL)*	27	TCTTTCTTACTTTACTTGTTGCTTGTC
	IGS *(psbT, psbN)*	3	IGS *(psbT, psbN)*	21	TTGAAGTAATGAGTCCCCCAA
	Intron *(petB, petB)*	9	Intron *(petB, petB)*	20	TAAATCGAAAGAAAGGTTTG

[1] IGS, intergenic spacer.

Table S7: List of *A. polymorpha* and *L. officinale* accessions used in this study.

No.	Species	Collection information	Voucher number	Coordinates	GenBank accession number	Marker test
1		Ilcheongungjaebaeji, Bonghwa-gun, Gyeongsangbuk-do, Korea	KIOM201501014665	36°48'01.9"N 128°57'47.2"E		o
2		Subi-myeon, Yeongyang-gun, Gyeongsangbuk-do, Korea	KIOM201701018797	36°47'39.8"N 129°12'19.6"E		o
3		Subi-myeon, Yeongyang-gun, Gyeongsangbuk-do, Korea	KIOM201701018796	36°47'39.8"N 129°12'19.6"E		o
4		Yeongyang-eup, Yeongyang-gun, Gyeongsangbuk-do, Korea	KIOM201501014665	36°40'32.0"N 129°06'46.2"E		o
5		Yeongyang-eup, Yeongyang-gun, Gyeongsangbuk-do, Korea	KIOM200601000207	36°40'32.0"N 129°06'46.2"E		o
6		Yeongyang-eup, Yeongyang-gun, Gyeongsangbuk-do, Korea	KIOM200601000208	36°40'32.0"N 129°06'46.2"E		o
7	*L. officinale*	Subi-myeon, Yeongyang-gun, Gyeongsangbuk-do, Korea	KIOM201701018769	36°47'39.8"N 129°12'19.6"E	NC039760	o
8		Subi-myeon, Yeongyang-gun, Gyeongsangbuk-do, Korea	KIOM201701018770	36°47'39.8"N 129°12'19.6"E		o
9		Subi-myeon, Yeongyang-gun, Gyeongsangbuk-do, Korea	KIOM201701018771	36°47'39.8"N 129°12'19.6"E		o
10		Subi-myeon, Yeongyang-gun, Gyeongsangbuk-do, Korea	KIOM201701018795	36°47'39.8"N 129°12'19.6"E		o
11		Subi-myeon, Yeongyang-gun, Gyeongsangbuk-do, Korea	KIOM201701018617	36°47'39.8"N 129°12'19.6"E		o
12		Subi-myeon, Yeongyang-gun, Gyeongsangbuk-do, Korea	KIOM201301005868	36°47'39.8"N 129°12'19.6"E		o
13	*A. polymorpha*	Wonju-si, Gangwon-do, Korea	KIOM201501014664	37°20'17.6"N 128°02'27.5"E	MH260705	o
14		Seoha-myeon, Hamyang-gun, Gyeongsangnam-do, Korea	KIOM201501015521	35°38'39.7"N 127°44'12.2"E		o

15	Yeonpung-myeon, Goesan-gun, Chungcheongbuk-do, Korea	KIOM201701020170	36°42'55.1''N 128°03'07.5''E	o
16	Changjuk-dong, Taebaek-si, Gangwon-do, Korea	KIOM201701020255	37°13'50.7''N 128°56'17.1''E	o
17	Wicheon-myeon, Geochang-gun, Gyeongsangnam-do, Korea	KIOM201501015376	35°42'53.8''N 127°47'45.7''E	o
18	Gohan-eup, Jeongseon-gun, Gangwon-do, Korea	KIOM201501015172	37°12'14.3''N 128°54'31.1''E	o
19	Dunnae-myeon, Hoengseong-gun, Gangwon-do, Korea	KIOM201001003015	37°29'28.6''N 128°14'52.1''E	o
20	Jinbu-myeon, Pyeongchang-gun, Gangwon-do, Korea	KIOM200901002083	37°47'58.0''N 128°32'35.2''E	o
21	Jinbu-myeon, Pyeongchang-gun, Gangwon-do, Korea	KIOM200901002084	37°47'58.0''N 128°32'35.2''E	o

Table S8: List of primers used for the validation of the CP genome sequence of *A. polymorpha*.

Primer name	Primer sequence (5′→3′)	Junction
LIGLF	GGATCCTCGCGGACAGAAAA	LSC/IRa
LIGLR	CGTGTCTGGTACTGCATGGT	
LIGISF	ACAATTCTAACTAGCCCTAATGGTC	IRa/SSC
LIGISR	TCAAATCTGTAGGTAGTGGGCG	
LIGSIF	ACGCCTTTGAACACGCATAG	SSC/IRb
LIGSIR	AGAGGTTGAACAGAAAATAGACCG	
LIGILF	GATCTGCAGGGTCCCAAATGA	IRb/LSC
LIGILR	ACTTTGATCCAACGGAGCCC	

Table S9: List of CP genomes downloaded from NCBI for phylogenetic analysis.

Order	Family	No.	Taxon	GenBank accession number
Apiales	Apiaceae	1	*Angelica acutiloba*	NC029391.1
		2	*Angelica dahurica*	NC029392.1
		3	*Angelica decursiva*	KT781591.1
		4	*Angelica gigas*	NC029393.1
		5	*Angelica nitida*	MF594405.1
		6	*Arracacia xanthorrhiza*	KY117235.1
		7	*Glehnia littoralis*	KT153022.1
		8	*Ledebouriella seseloides*	KT153021.1
		9	*Peucedanum insolens*	NC033344.1
		10	*Seseli montanum*	KM035851.1
		11	*Ligusticum tenuissimum*	NC029394.1
		12	*Ligusticum officinale*	NC039760.1
		13	*Daucus carota*	NC008325.1
		14	*Anthriscus cerefolium*	NC015113.1
		15	*Tiedemannia filiformis* subsp. *greenmannii*	HM596071.1
		16	*Coriandrum sativum*	NC029850.1
		17	*Bupleurum latissimum*	NC033346.1
		18	*Bupleurum falcatum*	NC027834.1
		20	*Anethum graveolens*	NC029470.1
		21	*Petroselinum crispum*	NC015821.1
		22	*Foeniculum vulgare*	NC029469.1
	Araliaceae	23	*Eleutherococcus senticosus*	JN637765.1
		24	*Fatsia japonica*	NC027685.1
		25	*Kalopanax septemlobus*	NC022814.1
		26	*Metapanax delavayi*	NC022812.1
		27	*Schefflera delavayi*	NC022813.1
		28	*Aralia undulata*	NC022810.1
		29	*Panax ginseng*	AY582139.1
		30	*Panax notoginseng*	NC026447.1
Dipsacales	Adoxaceae	31	*Adoxa moschatellina*	NC034792.1
		32	*Tetradoxa omeiensis*	NC034793.1
